# Platelet-Rich Plasma Effectiveness in Treating Androgenetic Alopecia: A Comprehensive Evaluation

**DOI:** 10.7759/cureus.77371

**Published:** 2025-01-13

**Authors:** Rúben Lopes-Silva, Maria Santos, Maria Luísa Sequeira, Andreia Silva, Tatiana Antunes, Paulo Valejo-Coelho, Manuel Neiva-Sousa

**Affiliations:** 1 Department of Maxillofacial Surgery, Unidade Local de Saúde de São José, Lisbon, PRT; 2 Department of Head and Neck Surgery, Instituto Português de Oncologia de Lisboa Francisco Gentil, Lisbon, PRT; 3 Functional Unit of Maxillofacial Surgery, Unidade Local de Saúde Alto Ave, Guimarães, PRT; 4 School of Medicine and Biomedical Sciences, Universidade Fernando Pessoa, Porto, PRT

**Keywords:** aga, androgenetic alopecia, hair follicle, platelet-rich plasma, prp

## Abstract

Platelet-rich plasma (PRP) has gained recognition in regenerative medicine due to its concentration of growth factors that promote hair follicle activity, making it a potential treatment for androgenetic alopecia (AGA). However, variability in PRP preparation and application has led to inconsistent outcomes across studies. This review evaluates the overall effectiveness of PRP in treating AGA based on the latest available evidence. A systematic search was conducted in PubMed, identifying 156 articles related to PRP and AGA. After applying inclusion and exclusion criteria, 11 studies published between January 2020 and May 2024 were selected. The studies focused on the efficacy of PRP compared to placebo or other treatments and explored different PRP formulations and application methods. The majority of studies demonstrated that PRP is effective in increasing hair density and thickness in patients with AGA. There was a general consensus on the positive effects of PRP, although results varied due to differences in preparation protocols, platelet concentration, and delivery methods. Additionally, combining PRP with other treatments, such as microneedling or topical medications like minoxidil, showed enhanced efficacy in several studies. While some studies reported conflicting outcomes, the overall evidence supports PRP as a promising treatment for AGA. In conclusion, PRP is a viable therapeutic option for AGA, particularly for increasing hair density and thickness. However, the variability in treatment protocols highlights the need for standardized PRP preparation and application methods. Future research should focus on refining these protocols and exploring the potential of combination therapies to maximize treatment effectiveness and consistency.

## Introduction and background

Platelet-rich plasma (PRP) is a potent tool in regenerative medicine, including hair restoration. Its composition encompasses a rich variety of biological components such as platelets, growth factors (GFs), cytokines, and other bioactive proteins [1,2]. Notably, among growth factors, basic fibroblast growth factor (bFGF) plays a critical role. bFGF stimulates the proliferation of dermal papilla cells, which are pivotal in regulating the growth and differentiation of hair follicles, helping to maintain them in the anagen phase [3]. The angiogenic properties of bFGF are especially advantageous in cases of androgenetic alopecia (AGA), where reduced blood flow to the scalp contributes to hair follicle miniaturization and subsequent hair loss [3,4]. Additionally, PRP can modulate cellular survival mechanisms by upregulating the expression of anti-apoptotic proteins while downregulating pro-apoptotic factors, thus enhancing the overall survival and function of hair follicles [5].

AGA is a common form of hair loss that affects both men and women and is characterized by progressive thinning of scalp hair. This condition is primarily driven by genetic predisposition and the action of androgens, particularly dihydrotestosterone (DHT) [6]. DHT binds to androgen receptors within hair follicles, leading to a progressive shortening of the anagen phase and an elongation of the telogen phase [7,8]. AGA can have a significant psychological impact, affecting personal, social, and professional aspects of individuals' lives [9,10].

Currently, treatments for AGA include drugs such as minoxidil, finasteride, and dutasteride, as well as hair transplantation surgery, microneedling, and PRP therapy. Combining these therapeutic options may offer synergistic benefits [11,12]. PRP, in particular, stands out due to its minimal adverse effects, which are generally limited to transient pain, mild erythema, and swelling at the application site, rendering it a safe treatment option [13]. PRP can be applied in either its activated or non-activated form, depending on the specific treatment protocol [14].

Nevertheless, despite its potential, the variability in PRP preparation techniques, the number of treatment sessions, the intervals between sessions, and the follow-up periods contribute to the inconsistent results observed across clinical studies [1].

This review seeks to provide a comprehensive analysis of PRP's efficacy in treating AGA, drawing upon recent scientific publications. Through a critical examination of the current body of evidence, this review aims to clarify the underlying mechanisms, clinical efficacy, and safety of PRP treatment for AGA while addressing the inconsistencies and knowledge gaps in the existing literature.

## Review

Materials and methods

The review was performed following the Preferred Reporting Items for Systematic Reviews and Meta-Analyses (PRISMA 2020) statement [15].

The PICOTS format was applied for the review selection process: population (P): female and male participants at the age of 18 years or older submitted to PRP injections to treat AGA; intervention (I): PRP injection in the scalp; comparison (C): placebo, platelet-rich fibrin (PRPF), and minoxidil; outcome (O): the primary outcomes focused on hair characteristics, such as hair density and hair thickness, the secondary outcomes focused on clinical and self-assessed evaluations; time (T): no specific follow-up period; study setting (S): randomized trials published between January 2020 and May 2024. Exclusion criteria were the inclusion of pediatric populations, application of PRP in other types of alopecia besides androgenetic (e.g., alopecia areata), other applications of PRP unrelated to alopecia, articles written in languages other than English, and publications outside the specified timeframe, conference abstracts, letters, or notes.

A systematic literature search was conducted in MEDLINE (PubMed) to identify recent articles addressing the use of platelet-rich plasma (PRP) in androgenetic alopecia (AGA). The query box ("PRP" OR "Platelet-Rich Plasma") AND ("AGA" OR "Androgenetic Alopecia") was used.

The first round of study selection involved analyzing article titles based on inclusion criteria. In the second round, articles were selected through abstract screening. Finally, the remaining articles were downloaded, and their full texts were evaluated to verify inclusion. The selection was performed independently by two reviewers, both authors of this paper (Lopes-Silva and Santos), and disagreements were resolved by brainstorming between them.

Results

**Figure 1:**
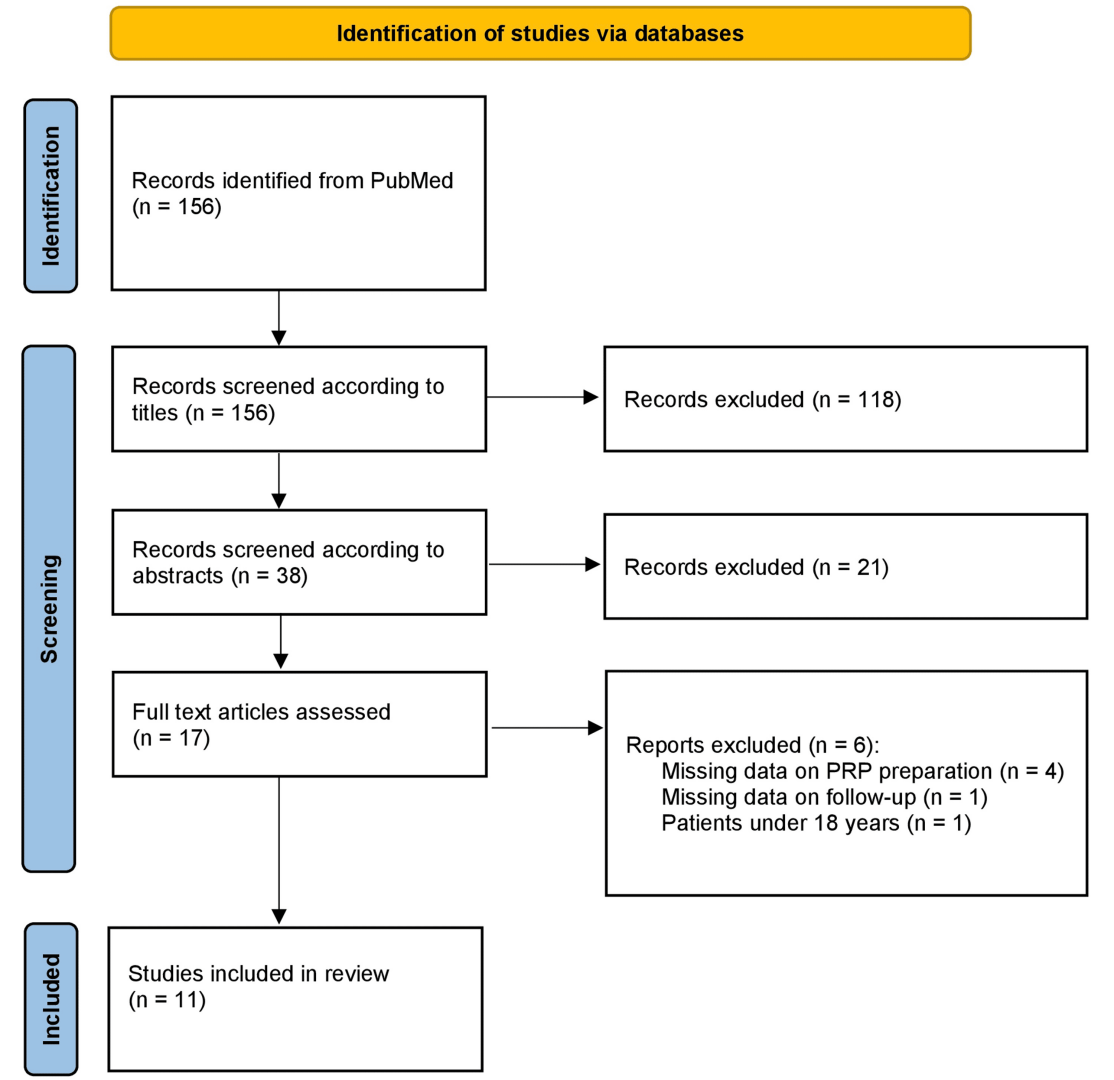
PRISMA flow diagram illustrating the study selection process, including the number of records identified, screened, excluded, and shortlisted for the final review. PRISMA: Preferred Reporting Items for Systematic Reviews and Meta-Analyses; PRP: platelet-rich plasma

The search in MEDLINE (PubMed) using the specified timeframe and keyword combination retrieved a total of 156 articles (Figure 1). After screening the corresponding titles, 38 articles and their abstracts were further analyzed to arrive at 17 papers for full-text evaluation. Of these 17 studies, six were excluded due to missing data on PRP preparation protocols, follow-up, and patient age. Finally, 11 articles were selected, comprising a total of 684 participants (514 men and 170 women), for detailed analysis.

Of the 11 studies, six (55%) exclusively investigated the efficacy of PRP alone for the treatment of AGA. One study (9%) examined the influence of platelet count and activation on treatment outcomes, while another (9%) evaluated the effectiveness of PRP administered point-to-point via syringe versus in conjunction with microneedling. The remaining five articles (45%) compared the efficacy of PRP in combination with other treatments. Specifically, one article (9%) compared PRP with plasma plus basic fibroblast growth factor for AGA treatment, two articles (18%) compared PRP with topical minoxidil, another (9%) investigated PRP in combination with minoxidil and either spironolactone or finasteride, and one (9%) examined PRP in conjunction with basic fibroblast growth factor and minoxidil.

To verify the efficacy of PRP in treating AGA, several studies have been designed to compare this autologous substrate with placebo groups (Table 1) [16-21].

**Table 1:** Articles included for comparing platelet-rich plasma (PRP) over the placebo. PRP: platelet-rich plasma; AGA: androgenetic alopecia

Author	Year	n	Study design	Outcome	Results
Dicle et al. [16]	2020	25	Two randomized groups receiving either monthly PRP or placebo for three sessions. After a three-month washout period, treatments were switched. Participants had six PRP sessions and were evaluated at baseline, four months, and nine months.	The outcome measured was hair density using trichoscopy. To ensure accuracy, evaluations were conducted by two dermatologists.	A significant increase in hair density was observed in the group who began receiving PRP administrations after the three-month washout period.
Gressenberger et al. [17]	2020	28	Two groups. One group received five treatments of 3-4 ml of PRP delivered intracutaneously, while the other group received saline solution.	The main outcome measures were hair density and hair diameter, both measured using the trichoscopy. The secondary objective was the clinical improvement, which was evaluated by an independent reviewer using patient photographs.	The change in hair density was not statistically different between the PRP-treated group and the control group. Hair thickness did not show statistically significant differences between the groups.
Ozcan et al. [18]	2022	62	Two groups of 31 participants. One group received four PRP sessions applied via dermapen microneedling at two-week intervals. The other group received PRP injected intraepidermally using a manual point-by-point technique with an insulin needle.	Outcomes: hair count, hair density, anagen hair, telogen hair, average hair length, vellus hair density, vellus hair count, terminal hair count, vellus hair ratio, terminal hair ratio. Hair pull tests and trichoscopy evaluations were conducted before and after treatments.	PRP treatment significantly improved hair pull tests, satisfaction scores, and increased hair count, density, terminal hair density, terminal hair count, and hair length compared to baseline in both treatment groups.
Qu et al. [19]	2021	52	Split-head study. In three consecutive sessions at one-month intervals, PRP was injected subdermally into half of the alopecia areas while the other half received saline. Global photographs were taken at baseline, three, and six months.	Outcomes: Hair count, hair density, hair diameter, anagen hairs. Photographic analysis was conducted by five experts.	After three PRP treatments, hair count, density, and diameter significantly improved at three and six months. PRP increased hair density from three months and hair count, diameter, and anagen hair ratio at six months compared to the control side.
Shapiro et al. [20]	2020	35	Split-head study. One side of the head received PRP while the other side received the same amount of saline solution. Participants underwent three treatment sessions at one-month intervals, with a final follow-up three months after the last session.	Primary outcome was hair density evaluation in AGA patients treated with non-activated PRP, compared to placebo, using trichoscopy by a blinded investigator. Secondary outcomes included changes in hair diameter, safety, and treatment tolerance.	The increase in hair density and hair diameter was not significantly greater than the increase in the placebo group. Approximately half of the participants noticed some improvement. Pain was the most reported symptom.
Singh and Singh [21]	2023	80	Split-head study in two groups. In group one, PRP with an activator was injected into the right half of the scalp and PRP without an activator into the left half. Group two received the opposite. Patients were further categorized by platelet counts in their PRP.	Primary outcomes were hair density and hair thickness. Secondary outcomes were patient self-assessment and changes in the Norwood Hamilton scale.	Hair density and thickness increased with and without activator, with higher platelet counts boosting both. Activator improved density at four months and thickness at six. No significant change in Norwood Hamilton scale. Patients showed moderate to marked improvement.

Shapiro et al. performed a randomized split-head study to assess hair density changes in AGA patients treated with standard non-activated PRP [20]. PRP was injected into one side of the scalp, while the other side received saline solution. Three treatment doses were administered one month apart, with a final follow-up visit three months after the third dose. The study found a statistically significant increase in hair density and diameter on the PRP-treated side compared to the placebo side. Additionally, 45.8% of patients reported a slight or noticeable improvement in the appearance of their scalp.

With the same purpose, Dicle et al. performed the study in two randomized groups [16]. Each group received three monthly sessions of PRP treatment or placebo (0.09% NaCl). After a three-month washout period, the groups switched treatments. Two dermatologists independently evaluated the captured images. At the end of the study, a significant increase in mean total hair count was observed in both groups. This increase was statistically significant between the fourth and ninth months for the group that received PRP in the second phase. However, there was no statistically significant difference between the baseline and the fourth month after PRP injections when compared to placebo injections.

Qu et al. conducted a randomized, placebo-controlled, double-blind, split-head study [19]. PRP was injected into half of the alopecia areas at zero day, three months, and six months, while the other half received saline as a control. Global photographs were taken at baseline, three months, and six months. Five independent experts, blinded to treatment assignment, used a five-point scale to assess the macrographs (scale ranging from much worse to much better). The average score for the PRP-treated side was 4.36, compared to 2.15 for the placebo side. Patient satisfaction was also high, with a mean score of 4.23.

Ozcan et al. explored the optimal method of application, comparing microneedling with point-by-point injections [18]. In their randomized study of 62 patients, one group received four PRP sessions applied via dermapen microneedling, while the other group received PRP injections using a manual point-by-point technique. Both methods led to significant increases in hair count and density. Hair pull tests showed no significant difference between the groups post-treatment, with 87.1% of the microneedling group and 77.4% of the point-by-point group achieving negative results. Singh and Singh examined whether platelet concentration and PRP activation impacted the treatment outcomes [21]. In this randomized, double-blind, split-head study, PRP with and without an activator was injected into opposite sides of the scalp. Hair density and thickness were measured monthly for six months. Results showed significant improvement in hair density and thickness regardless of activator use, with the highest increase in density (42%) observed in the group with the highest platelet count (>10 lakh/mm³). The study concluded that higher platelet concentrations lead to better outcomes.

The study by Gressenberger et al. showed contrasting results [17]. In this study, no significant difference in hair density or thickness between PRP-treated and placebo groups was observed. Participants were randomized, with one group receiving PRP intracutaneously and the other saline solution. Five treatment sessions were conducted at monthly intervals, with follow-up visits at four weeks and six months. No statistically significant changes were observed between the treated and placebo groups at both time points.

Five studies have investigated whether PRP is more advantageous when compared to other substances or if its effectiveness in treating AGA can be enhanced with other treatment methods (Table 2) [22-26].

**Table 2:** Studies comparing PRP/PRPF with other treatments. PRP: platelet-rich plasma; PRPF: platelet-rich fibrin

Author	Year	n	Study design	Outcome	Results
Afzal et al. [22]	2024	70	Two groups of 35 participants. One group received monthly injections of PRP and the other 5% topical minoxidil therapy given as 1 ml, two times daily for six months. Participants of both groups were assessed before treatment and after three and six months.	The efficacy of both groups was evaluated using the patient satisfaction scores, global photography evaluated by a dermatologist, and the hair pull test.	At six months, 77% of PRP patients had a negative hair pull test vs. 40% with minoxidil. Improvement was reported by 91.4% in the PRP group and 74.3% with minoxidil. PRP was effective in 74.5% vs. 43.7% for minoxidil.
Balasundaram et al. [23]	2023	51	Two groups. The minoxidil one used 5% topical minoxidil twice daily for six months, while the PRP group received non-activated PRP injections over three monthly sessions, with a final follow-up three months post-treatment.	Primary outcomes evaluated by two blinded dermatologists: hair count, hair density, and anagen proportion Secondary objectives included patient satisfaction score, safety, and tolerance.	No statistical difference was seen between the groups in increasing total hair count, terminal hair count, and density at week 12. The median patient satisfaction score for hair texture at week 24 was better for minoxidil than PRP, but not for hair density.
Qu et al. [24]	2022	80	Split-head study in two groups. One group received injection of PRPF in the right side of the head and saline in the other side; the other group received injection of PRPF in the right side of the head and PRP in the other side. The treatment was processed three times, one month apart.	Outcomes: hair count, hair density, terminal/vellus hair amount, mean hair diameter, hair growth rate, telogen hair ratio, and global appearance. Patient satisfaction and side effects was recorded. Evaluations included trichoscopy, global photography, and hair pull tests.	The administration of PRPF showed a statistically significant improvement on hair loss compared to placebo. PRPF seems to be superior to PRP alone on increasing hair count. Satisfaction evaluation scored an average of eight (one to ten scale) and the side effects were minimal.
Ramadan et al. [25]	2021	126	Three groups participated: two received PRP via microneedling or injections, while the control group did not. All received 5% topical minoxidil; women also took 100 mg spironolactone, and men took 2.5 mg finasteride, with PRP given over three to six months.	Outcomes focused on enhancing hair density and comparing the changes in AGA grades before and after therapy, assessed by three dermatologists using dermoscopy. Patient satisfaction was evaluated.	PRP treatment significantly improved outcomes compared to control, with microneedling outperforming syringe injections. After six months, 95% of patients had negative pull tests, and hair diameter and density increased more in the microneedling group than in the other group.
Wu et al. [26]	2023	75	Three groups. The first group received intradermal PRPF injections. The second group received topical minoxidil 5% twice a day. The third group received a combination of minoxidil and intradermal PRPF injections. Groups one and two received three PRPF treatment sessions at one-month intervals.	Hair count, terminal hair count, vellus hair ratio, hair density, mean thickness, hair growth rate, telogen hair ratio, and global appearance evaluated with trichoscopy and global photographs. Secondary outcomes: patient satisfaction with the treatment and side effects.	A notable increase in hair count, density, and growth rate was noted following PRPF therapy compared to minoxidil treatment. PRPF combined with topical minoxidil yielded greater enhancements in efficacy and patient satisfaction compared to monotherapy.

Two studies compared PRP with minoxidil. Balasundaram et al. found that both treatments led to significant increases in basal hair count, hair density, terminal hair count, and density at week 12, with no significant difference between groups [23]. Conversely, Afzal et al. noted that 77% of patients treated with minoxidil had a negative hair pull test, compared to only 40% in the PRP group [22]. While patient satisfaction with hair texture favored minoxidil at week 24, there were no significant differences in hair density.

In order to evaluate whether basic fibroblast growth factor (bFGF) released by platelets stimulates dermal papilla cell proliferation, Qu et al. investigated whether bFGF at different concentrations could affect outcomes [24]. They divided patients into groups receiving PRP or PRPF (PRP with added bFGF) via a split-head design study. PRPF showed a statistically significant improvement in hair count, density, terminal hairs, and anagen hairs compared to PRP alone, especially at one-, three-, and six-month follow-up points.

Wu et al. compared PRPF to minoxidil and found that monotherapy with PRPF was more effective in increasing hair count and density [26]. Combination therapy of PRPF with minoxidil was even more effective than either treatment alone. However, hair thickness did not differ significantly between the groups. Terminal hair count increased in both the PRPF and combination therapy groups, whereas vellus hair ratios decreased after six months.

Ramadan et al. compared different PRP application methods and combined treatments [25]. The study enrolled 126 participants divided into three groups. One group served as a control, while the other two received PRP via syringe or following microneedling. All patients received topical minoxidil and either spironolactone (for women) or finasteride (for men). After six months, the group treated with microneedling showed significantly greater improvements in hair diameter and density than the syringe group. Overall, 88% of the patients were satisfied with the treatment results.

In all these studies, pain was commonly reported as the main complaint. However, no major adverse effects were described in any of the trials [16-26].

Discussion

The reviewed studies provide a comprehensive assessment of the efficacy of platelet-rich plasma in treating androgenetic alopecia, highlighting its potential to improve hair density and thickness. Several placebo-controlled trials offer compelling evidence that PRP significantly enhances hair density and diameter compared to placebo treatments [16,19,20]. The efficacy of PRP is attributed to its high concentration of growth factors, including PDGF, TGF-β, and VEGF, which stimulate hair follicle activity and promote the anagen (growth) phase of the hair cycle [19].

However, Gressenberger et al. reported contrasting results, observing no significant difference in hair density or thickness between the PRP-treated and placebo groups [17]. Similarly, Afzal et al. and Balasundaram et al. conducted studies comparing the effectiveness of PRP and minoxidil [22,23]. Both studies presented conflicting conclusions, with Balasundaram et al. finding no significant difference between the two treatments in terms of hair count and density, while Afzal et al. reported that minoxidil performed better than PRP in the hair pull test. These discrepancies across studies may stem from variability in PRP preparation methods, including the number of centrifugation steps (single versus double spin), the final platelet concentration, and the use of activators, as well as differences in application techniques, such as microneedling versus point-by-point injections, or even inherent differences in response of study subjects towards treatment (Table 3).

**Table 3:** Variability in platelet-rich plasma (PRP) preparation and application protocols in reviewed studies. * three sessions at two-week intervals and the fourth session one month after the last session; ** no precise information was obtained.

Author	Collected blood (ml)	Centrifugation	PRP obtained (ml)	PRP applied (ml/cm2)	Sessions’ number	Sessions’ intervals	Follow-up (months)
Alzal et al. [22]	30	Double Spin	-	0.1-0.2	6	1 month	6
Balasundaram et al. [23]	19	Double Spin	2	0.1–0.2	3	1 month	6
Dicle et al. [16]	30	Single Spin	5	-	3	1 month	9
Gressenberger et al. [17]	20	Single Spin	3-4	0.1	5	4-6 weeks	6
Qu et al. [19,24]	40	Double Spin	4	0.05-0.1	3	1 month	6
Ozcan et al. [18]	10	Single Spin	4-5	0.1	4	2 weeks^*^	6-12^**^
Ramadan et al. [25]	10	Single Spin	5	0.1	3-6	1 month	9
Shapiro et al. [20]	10	Single Spin	5	0.1-0.2	3	1 month	6
Singh and Singh [21]	25	Double Spin	2	0.1	3	1 month	6
Wu et al. [26]	-	-	1	0.05-0.1	3	1 month	6

The preparation of PRP is a critical factor influencing its therapeutic effects, and the lack of standardization in PRP preparation protocols confounds the comparison of results across studies [17,18]. Some studies used non-activated PRP, while others explored the use of activated PRP, which involves pre-treatment with agents that induce the platelets to release growth factors before injection [19-21]. Singh et al. demonstrated that higher platelet counts and activation significantly improved hair density and thickness over time, suggesting that platelet activation might play a pivotal role in enhancing PRP's effectiveness. Still, activation methods vary, and further research is needed to determine the optimal activation protocol and maximize clinical outcomes.

In addition to the variability in preparation and activation, the method of application has emerged as a key factor in the success of PRP treatments. Ozcan et al. investigated the efficacy of PRP delivered via microneedling versus point-by-point injections [22]. They found that microneedling was superior in promoting hair growth, with patients in the microneedling group showing higher hair counts and density compared to those receiving injections. This suggests that microneedling may enhance PRP's therapeutic effects by increasing the surface area of absorption and stimulating the scalp, potentially leading to more effective growth factor delivery to the hair follicles. These findings indicate the need for further studies comparing different application methods to optimize treatment protocols.

The introduction of platelet-rich fibrin (PRPF) offers another avenue for enhancing PRP's effectiveness. Qu et al. conducted a split-head study comparing PRPF and PRP, revealing that PRPF-treated areas had significantly higher hair counts, density, and terminal hair growth compared to PRP-treated areas [24]. PRPF contains a higher concentration of growth factors, particularly basic fibroblast growth factor (bFGF), which has been shown to stimulate the proliferation of dermal papilla cells, a critical component in hair follicle development and cycling [24]. These findings underscore the importance of growth factor concentration in determining treatment efficacy. Similarly, Wu et al. evaluated the efficacy of PRPF in combination with minoxidil and found that the combination therapy led to greater improvements in hair count, density, and growth rate compared to that in PRPF or minoxidil monotherapy [26]. This suggests that PRPF may offer a more potent formulation of PRP, enhancing its ability to stimulate hair growth.

Combination therapies involving PRP or PRPF with other treatments have shown promise in further enhancing treatment efficacy [22-26]. Ramadan et al. investigated the effects of combining PRP with microneedling, minoxidil, and finasteride or spironolactone in AGA patients [25]. Their results indicated that combining PRP with microneedling leads to significantly greater increases in hair diameter and density compared to PRP injections alone. Furthermore, 88% of patients expressed high satisfaction with the outcomes, indicating that combining PRP with other therapeutic interventions can significantly improve clinical results. Similarly, Wu et al. found that combining PRPF with topical minoxidil yielded superior results compared to their single treatment, which further supports the idea that synergistic effects can be achieved through combination therapies [26].

Another important consideration is the timing and frequency of PRP treatments. Studies reviewed in this discussion varied widely in their treatment protocols, with some delivering PRP in three to six monthly sessions, while others applying PRP at shorter intervals, such as every two weeks [18,20,21]. The interval between treatments and the duration of follow-up appear to play significant roles in determining the long-term efficacy of PRP therapy. For example, Qu et al. observed that the effects of PRP continued to improve over a six-month period, suggesting that multiple sessions with extended follow-up are necessary to achieve optimal results [19]. Standardizing the frequency of treatment sessions and follow-up periods could help achieve more consistent results across studies.

## Conclusions

Despite the largely positive findings regarding PRP's efficacy, the variability in patient responses underscores the complexity of its therapeutic potential for AGA. While most of the evaluated studies have demonstrated marked improvements in hair density and thickness for many patients, others reported minimal or no benefits. This inconsistency may stem from a range of factors, including individual variations in hair follicle biology, the progression stage of AGA at the time of treatment, and genetic predispositions affecting responsiveness to growth factors. Furthermore, the influence of the placebo effect cannot be disregarded, emphasizing the subjective nature of patient self-assessments. Addressing these variations requires deeper exploration into the biological determinants of PRP responsiveness and the development of more tailored therapeutic strategies.

While PRP offers significant promise as a treatment for AGA, the heterogeneity in protocols and patient outcomes indicates a pressing need for further research. Efforts to standardize the preparation and application methods of PRP could enhance the reliability of clinical results. Moreover, investigating the potential of combination therapies may open new avenues to optimize patient outcomes. As the understanding of PRP therapy evolves, a more personalized and evidence-based approach could help bridge the gap between its promising potential and the current challenges in achieving consistent treatment success.
